# Ideation and implementation of an open science drug discovery business model – M4K Pharma

**DOI:** 10.12688/wellcomeopenres.14947.1

**Published:** 2018-12-06

**Authors:** Maxwell Robert Morgan, Owen Gwilym Roberts, Aled Morgan Edwards

**Affiliations:** 1 University of Toronto, Toronto, ON, M5G 1L7, Canada; 2 M4K Pharma, Toronto, ON, M5G 1L7, Canada; 3 Structural Genomics Consortium, London, UK

**Keywords:** Open science, open drug discovery, rare diseases, regulatory exclusivity

## Abstract

M4K Pharma was incorporated to launch an open science drug discovery program that relies on regulatory exclusivity as its primary intellectual property and commercial asset, in lieu of patents.In many cases and in key markets, using regulatory exclusivity can provide equivalent commercial protection to patents, while also being compatible with open science. The model is proving attractive to government, foundation and individual funders, who collectively have different expectations for returns on investment compared with biotech, pharmaceutical companies, or venture capital investors.In the absence of these investor-driven requirements for returns, it should be possible to commercialize therapeutics at affordable prices.M4K is piloting this open science business model in a rare paediatric brain tumour, but there is no reason it should not be more widely applicable.

## The drug development business model

The discovery of new medicines is increasingly expensive and risky ^1– 5^, and the business model has become predicated on the pricing of new medicines at levels barely manageable by even affluent countries ^6– 8^. Over the years, and in an effort to improve the situation, the main players in the ecosystem - academia, industry, governments, foundations, and patient groups - have been exploring new models of collaboration, and new schemes for funding and rewarding drug discovery. For example, recent years have seen an explosion of academic drug discovery efforts ^9, 10^. However, although the location of drug discovery activities has moved among the players, the fundamentals have not changed. The costs of discovery and the prices of new medicines continue to rise, but there has yet to be a transformative change in the business model. And there are consequences: there are substantially diminished research efforts in riskier or unprofitable areas of drug discovery, such as the neurosciences ^11^, anti-infectives ^12^, and tropical and paediatric diseases ^13– 15^. The root causes of the problem are manifold, but include the fact that the current drug discovery system is built on business models that emphasize, even require, proprietary generation and use of knowledge, which in turn leads to secrecy, needless duplication of effort, and ultimately inefficient use of human and financial capital ^3, 4^. Open science may provide a solution to this problem, and it is a model that we are piloting at M4K Pharma (M4K, for **M**eds for **K**ids). 

## Open science and the discovery of drug targets

As exemplified by the Structural Genomics Consortium ^16^, open science – the rapid multilateral sharing of knowledge, results, data, and materials without patent restrictions ^17^ – has proven to be tremendously successful in pre-competitive research areas related to early-stage drug discovery ^18^. Open science can: (i) lower transactional barriers to collaboration, (ii) encourage cross-disciplinary contributions of expertise, (iii) distribute project risk, (iii) reduce redundancy, (iv) enable more rapid generation of new hypotheses, (v) enable transparent peer review, and (vi) increase reproducibility ^3, 17– 19^.

Our hypothesis is that such an open organizational framework can be successfully applied not only to accelerate basic science but also to advance an innovative new drug candidate through discovery, preclinical and clinical development, regulatory approval, and health system uptake. Expanding the scope of open science to include more aspects of drug discovery would amplify its impact by: (i) permitting secondary and meta-analyses to improve decision-making by researchers, funders, health regulators, payers, prescribers, and patients; (ii) providing a mechanism to share failed projects and trials; and (iii) better respecting clinical trial participants by maximizing the scientific benefits of their generous contributions while minimizing their exposure to risk in duplicative studies ^4, 19^.

## Application of open science to drug discovery and development

Although open science has the potential to create a far more efficient drug discovery ecosystem, it has proven difficult to apply to individual drug discovery and development programs. The greatest concern is that practicing open science makes it more challenging to manage and protect intellectual property: open science creates prior art in the public domain and also distributes inventorship among scientists in many institutions, potentially without legal agreements in place. The problem is that these properties of open science are inconsistent with creating a patent position, which is the most common intellectual property tool to shield a new drug from generic competition. In fact, it is widely believed that patenting is not only important, but is actually essential to incentivize drug development ^20, 21^. This is not the case. As detailed in the next section, sponsors of newly approved medicines in most commercially important jurisdictions are also granted other powerful intellectual property protections in the form of regulatory data and market exclusivities ^22^. These protections are granted whether the drug product is patented or not, as well as provide better, and sometimes longer, barriers to entry from generic competition. In essence, these protections offer a strong alternative to patents for incentivizing drug development and commercialization ^23– 25^ and allow for an open science approach to drug discovery and development. 

## Regulatory exclusivity - a powerful form of intellectual property

Many governments, through their regulatory mechanisms for drug approval, provide an array of non-patent-based incentives to stimulate the discovery of new medicines, and to protect sponsors of new drugs from competition. 

The most common form of incentive is regulatory data protection for drugs containing new active ingredients (this is often referred to as *new chemical entity (NCE) exclusivity* for small molecule drugs), in which regulators grant the drug sponsor exclusive rights to the preclinical and clinical data they used to gain regulatory approval for periods of time. This form of “regulatory exclusivity” is valuable because it blocks generic competition: without the ability to reference these data, generic companies are unable to use the abbreviated drug approval mechanisms offered by regulators (e.g. the Abbreviated New Drug Application (ANDA) mechanism in the US). The period of exclusivity varies depending on the product (small molecule or biologic) and among jurisdictions, but the period is significant (for example, 10 years in the EU) ^22^ and constitutes valuable intellectual property. With respect to open science, many major drug product markets (including the US, EU, Switzerland, Canada, Israel, Japan, South Korea, Singapore, and Taiwan) apply new chemical entity exclusivity even if the sponsor’s data are publicly available ^22, 26^.

Several governments (US, EU, Singapore, Japan, Australia, Taiwan, and South Korea), through their drug regulators, offer an additional form of regulatory exclusivity (called *orphan drug exclusivity*) for drugs approved for rare diseases, regardless of whether the drugs contain new active ingredients. For these drugs, which must be first granted “orphan status” designation, no competitor may market the same active ingredient in the same rare disease indication, even in the unlikely circumstance that the competitor were to generate its own complete regulatory data package ^27, 28^.

There are additional regulatory exclusivity incentives for other special cases, including: data protection for new clinical studies of previously approved active ingredients in the US; exclusivity extensions for paediatric studies in the US, EU, and Canada; exclusivity extensions for new indications in the EU; and exclusivity extensions for new antimicrobial drugs to treat serious or life-threatening infections through the Generating Antibiotic Incentives Now (GAIN) Act in the US ^29, 30^.

Regulators also provide non-exclusivity-based incentives for drug development in specific under-served markets. For example, the US offers “priority review vouchers” to sponsors who achieve new drug registrations for tropical diseases and rare paediatric diseases. These vouchers permit their owners to accelerate regulatory approval of any subsequent drug product. Interestingly, these can be auctioned in the secondary market and have generated as much as USD $350 million ^31^, though prices have fallen to the USD $110 to $130 million range in 2017 and 2018 ^32^.

## Regulatory exclusivity compares favourably with patent protection

A patent grants its owner 20 years of exclusive use of the claimed invention. However, the core “composition of matter” patent of an innovative drug most often yields only 8–12 years of exclusive marketing rights, even after patent term restoration, due to the length of the discovery, clinical trial, and approval processes ^33^. To extend their marketing exclusivity and to create further barriers to generic competition, companies often adopt an intellectual property strategy that involves filing additional patents on polymorphs, formulations, and dosage forms. This strategy is very costly and these types of patents are frequently invalidated in litigation ^34, 35^. Nevertheless, patents remain the mainstay mechanism through which innovative drug companies attempt to exclude competitors from the market. 

**Figure 1.**
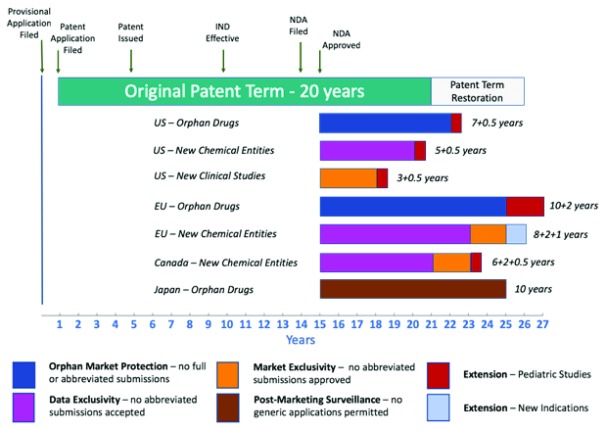
Comparing protection against competition for a new drug sponsor under an average effective patent term in the US with protection against competition under prospective M4K regulatory exclusivity periods in the US, EU, Canada, and Japan, using a new drug targeting DIPG as the exemplar. The average effective composition of matter patent term for a new drug after patent restoration in the US is approximately 11–12 years ( *source*: Cárdenas-Navia, J. Thirty Years of Flawed Incentives: an Empirical and Economic Analysis of Hatch-Waxman Patent-Term Restoration. *Berkeley Technol. Law J*. 29, (2015)). In comparison, irrespective of its patent status, a new drug approved to treat DIPG could be entitled to (i) orphan drug exclusivities of 7.5 years in the US (including a 6-month paediatric extension) and 12 years in the EU (including a 2-year paediatric extension); (ii) new chemical entity exclusivities of 5.5 years in the US (including a 6-month paediatric extension), 10 years in the EU, and 8.5 years in Canada (including a 6-month paediatric extension); and (iii) a period of orphan drug post-marketing surveillance of 10 years in Japan (which acts as an equivalent bar to entry by competitors). Approval of subsequent indications for the same drug could entitle M4K to (i) 3.5 years of new clinical study exclusivity in the US (including a 6-month paediatric extension, if the new indication required further paediatric studies) and (ii) a 1-year extension of new chemical entity exclusivity in the EU (for a total of 11 years).

The period of market exclusivity granted by the array of regulatory protections compares favourably with the average length of patent protection post-registration ( Figure 1). For example, a company that successfully registered an openly developed drug with a new active ingredient in the US, EU, Canada, and Japan would be entitled to: (i) new chemical entity (NCE) exclusivity for periods of 10 years in the EU, 8 years in Canada, and 5 years in the US; and (ii) 8 years of post-marketing surveillance protection in Japan (an equally effective barrier to generic competition) ^22^. If a company registered a drug for a rare paediatric disease in those markets, it would also be granted (i) concurrent orphan drug exclusivity in the rare disease indication of 7 years in the US and 10 years in the EU, (ii) paediatric extensions of an additional 2 years of orphan drug exclusivity in the EU (for a total of 12 years) and an additional 0.5 years of NCE exclusivity in Canada (for a total of 8.5 years), and (iii) 10 years of orphan drug post-marketing surveillance protection in Japan ^22, 27– 30^. If a company instead registered a new biologic in the US, the regulatory protections are even more favourable; it would be eligible for 12 years of exclusivity ^29^. If a company registered a new antimicrobial to treat a serious or life-threatening infection, it would gain a 5-year extension of US NCE exclusivity (for a total of 10 years of protection) ^29^.

These regulatory exclusivity incentives provide significant commercial advantages. Regulatory exclusivity periods, unlike patents: (i) are virtually costless to obtain, automatically enforced by regulators, and generally not subject to challenge by would-be competitors; (ii) can be obtained for compositions of matter or potential uses thereof that have been previously disclosed in public literature; and (iii) only begin once a drug receives marketing authorization, thereby providing a sponsor with *ex ante* certainty over the period of market protection ^23– 25, 36^.

## The use of regulatory incentives in the real world

The business case for relying on regulatory exclusivity is also bolstered by real world evidence. After the introduction of NCE protection in the US through the Hatch-Waxman Act, at least 26 drugs containing novel active ingredients were brought to market in the US reliant entirely on NCE exclusivity without listing any patents against the product in the FDA Orange Book ( Table 1) ^30, 37^. After orphan drug exclusivity was introduced in the US and EU in 1983 and 1999, respectively, there was significantly increased development efforts and product registrations to treat rare diseases in those jurisdictions - even though the laws had no impact whatsoever on available patent protections ^27^. Perhaps the greatest evidence of the commercial importance of regulatory exclusivities lies in the aggressive efforts by industry and trade representatives in the US and EU to negotiate expanded pharmaceutical data protections around the world ^38^.

**Table 1.** Examples of FDA new drug approvals from 1986 to 2014 brought to market with new chemical entity exclusivity but either (i) no patents listed in the FDA Orange Book, or (ii) listed patents expiring prior to new chemical entity exclusivity. The priority review eligibility and orphan drug status of each drug are also indicated. *Source:* Lietzan, E. The Myths of Data Exclusivity. *Lewis Clark Law Rev.* 20, 91–164 (2016).

Year of Approval	Drug	Indication	Priority Review Granted (‘Significant Improvement’ Over Standard of Care)	Concurrent Orphan Drug Exclusivity	Orange Book Listed Patent (Expiry)
1986	Provocholine (methacholine chloride)	Diagnosis of bronchial airway hyper-reactivity in patients who do not have clinically apparent asthma	+		
1987	Levatol (penbutolol sulfate)	Mild to moderate arterial hypertension			
1989	Anafranil (clomipramine hydrochloride)	Obsessive-compulsive disorder	+		
1989	Optipranolol (metipranolol hydrochloride)	Open-angle glaucoma and other causes of ocular high pressure			
1989	Lariam (mefloquine hydrochloride)	Mild to moderate acute malaria	+		
1989	Clorazil (clozapine)	Severely ill schizophrenic patients	+		
1990	Hexalen (altretamine)	Refractory ovarian cancer	+	+	
1993	Leustatin (cladribine)	Active hairy cell leukemia	+	+	
1993	Trasylol (aprotinin bovine)	Reduction of bleeding during complex surgery	+	+	
1993	Flumadine (rimantadine hydrochloride)	Influenza type-A infections	+		
1995	Revex (nalmefene hydrochloride)	Partial reversal of effects of narcotics			
1996	Proamatine (midodrine hydrochloride)	Orthostatic hypotension		+	
1997	Normiflo (ardeparin sodium)	Prevention of blood clot formation following certain types of surgery			
1997	Corlopam (fenoldopam mesylate)	Short-term management of hypertension			
1998	Infasurf (calfactant)	Respiratory distress syndrome in premature infants			
1999	Nilandron (nilutamide)	Treatment of prostate cancer in men who have undergone surgical castration			
1999	Curosurf (poractant alfa)	Respiratory distress syndrome in premature infants			
2000	Celexa (citalopram hydrobromide)	Depression			
2000	Innohep (tinzaparin sodium)	Deep vein thrombosis			
2003	Elestat (epinastine hydrochloride)	Prevention of itching associated with allergic conjunctivitis			
2004	Sanctura (trospium chloride)	Overactive bladder			
2011	Potiga (ezogabine)	Epileptic seizures			
2011	Firazyr (icatibant)	Hereditary angioedema	+	+	+ (July 2015)
2011	Ferriprox (deferiprone)	Iron overload in patients with thalassemia receiving blood transfusions		+	
2012	Choline C 11	PET scan imaging agent for detection of recurrent prostate cancer			
2013	Dotarem (gadoterate meglumine)	MRI contrast agent for use in brain and spinal tissues			
2014	Impavido (miltefosine)	Bacterial leishmaniasis		+	

## M4K Pharma – implementing an open science business model

M4K was founded to substantiate the commercial opportunity provided by regulatory data and market exclusivity protections for a new drug developed using open science. M4K aims specifically to discover and develop a precision medicine to treat a genetic subset of diffuse intrinsic pontine glioma (DIPG), an aggressive form of paediatric brain cancer with a small patient population and no effective therapeutic options. One quarter of DIPG tumours have an activating mutation in the ALK2 protein kinase ^39^. This has led to the hypothesis that an inhibitor of the ALK2 kinase will have therapeutic benefit in this subset of patients. 

Like all small companies, M4K faces scientific and business challenges. The scientific challenge is to create a potent, selective, safe, and efficacious drug that is brain penetrant. M4K is tackling this using a traditional structure-guided drug discovery and development scientific path. The business challenges are: to raise the funding to finance drug discovery and development; to create a strong intellectual property position that can be licensed to a drug manufacturer; and ultimately to be able to negotiate affordable pricing. M4K is tackling these using open science, and by adopting the following business strategies.

1. *A partnering strategy that encourages publication and data sharing*

The aim of the M4K discovery and development strategy is to align independent funding sources and a broad network of scientists towards its drug discovery aims, while both allowing and encouraging each participant to meet their own research objectives. For example, while partners who contribute funding to M4K, such as government and charitable organizations, are helping to invent a new medicine, they are also advancing their own organizational aims, be they knowledge generation or disease cures. While academic scientists and clinicians who collaborate with M4K are helping to contribute to the discovery of a new medicines, they are also advancing their academic careers, as M4K encourages any collaborating scientist to openly communicate or publish their findings. 

The M4K partnering strategy also allows industry to participate in M4K’s programs to mutual benefit. M4K gains directly from *pro bono* contributions from Contract Research Organizations (CROs), such as from Charles River Laboratories and Reaction Biology Corp, and the CROs in turn benefit by improving employee morale, by being able to openly showcase capabilities to other potential clients, by generating training opportunities, and by advancing corporate social responsibility. Even pharmaceutical companies have shown interest in participating. In addition to their altruistic motivations, M4K could also advance their business interests by helping achieve clinical validation of an interesting therapeutic target and potentially inventing a product to in-license. In summary, the M4K model provides a nexus of shared interests.

2. *A partnering strategy that develops an intellectual property position*

M4K encourages broad and rapid dissemination of its research results, and its partnership agreements include terms intended to codify open science into the relationship. For example, the agreement terms stipulate that none of the research activity related to M4K will be patented. Collaborators that carry out studies intended for regulatory submission will also need to agree that the exclusive right to use the underlying data for regulatory purposes is allocated to M4K. This restriction will not, however, inhibit public disclosure of the preclinical and clinical datasets, which will be released under a minimally restrictive click-wrap data use agreement that allows for broad follow-on research use but prohibits regulatory use without M4K authorization. M4K documents released in this manner will be clearly watermarked with these terms. The EMA has already begun releasing drug sponsors’ clinical data through an analogous mechanism ^40^.

These clear up-front positions may deter some scientists and institutions, but in our experience so far with M4K and the SGC, this is rare and the clarity of the commitment to sharing and affordable pricing will attract far more.

3. *M4K has a corporate structure inviting for scientists and public and charitable funders*

The open science business model depends on contributions from scientists in many institutions and from multiple public and philanthropic funders. In our view, the greatest barrier to attracting these contributions is the perception that one or more of M4K’s principals or other contributors would unfairly benefit. To eliminate this perception, and to ensure that all are treated equally, we structured M4K so that no executive, scientist, institution, or funder is entitled to equity or royalty payments. Instead, all equity in M4K is held by an arm’s length charity – the Agora Open Science Trust – which is governed by an independent board of directors and whose mandate is to use any proceeds from M4K to support open science and the public good. 

4. *Licensing strategy*

Without profit-driven ownership, M4K can adopt a licensing strategy that prioritizes affordable pricing instead of maximizing returns to the company. Accordingly, once M4K generates a clinical asset that is sufficiently de-risked, it intends to license the rights to its regulatory data package (as well as any marketing authorizations, regulatory exclusivities, and voucher incentives to which it is entitled) to one or more partners capable of bringing the medicine to patients. Because M4K’s asset will have been substantially de-risked through public and philanthropic contributions, M4K will seek to negotiate and enforce pricing concessions to ensure affordable access for patients. And if there are any net proceeds from licensing that accrue to M4K, they will be distributed to the Agora charity to further the public good. It is worth highlighting that affordable pricing licensing agreements of clinically de-risked assets to an industry partner have been successfully negotiated by the Medicines for Malaria Venture (MMV) and the Drugs for Neglected Diseases initiative (DNDi) on several occasions ^41– 43^.

This affordable licensing approach can be contrasted with that adopted by the Cystic Fibrosis Foundation (CFF) when it invested USD $75 million of its charitable funding into the development of ivacaftor, a targeted medicine for cystic fibrosis patients with certain gene variants. CFF sold its right to future royalties in 2014 for US $3.3 billion but did not seek to constrain pricing. As a result, Vertex Pharmaceuticals, which acquired the rights to ivacaftor, launched it at over USD $300,000 per patient per year ^7^.

## Experience after one year of M4K activities

M4K commenced operations in November 2017, and has since progressed its early-stage drug discovery program into lead optimization, with help from significant non-dilutive funding from public and philanthropic sources, as well as generous in-kind contributions of advice, materials, and research efforts from a range of participants. These contributions have reduced direct development costs and accelerated discovery. The progress on the ALK2 drug discovery project as of November 2018 can be viewed online.

1. *Funding of M4K activities*

M4K was incorporated to be well positioned to obtain financial support from nearly all biomedical research funding sources (governments, pharma, foundations, individuals, and institutions), with the possible exception of venture capital. Indeed, many governments are creating specific funding opportunities for drug discovery ^44^, and although most expect, and sometimes demand, that recipients protect their advances with patents, others are more open to other approaches (see here and here). 

M4K successfully competed for one such drug discovery grant – part of the Cancer Therapeutics Innovation Pipeline program from the Ontario Institute for Cancer Research (OICR). The funds (CAD$2M) are being used to support the direct and indirect costs of running M4K and a portion of the research being carried at OICR, the University of Oxford, and partner CROs. Additional funding from the Brain Tumour Charity to M4K’s academic collaborators at the University of Oxford is being used to support complementary scientific studies. M4K is well positioned to raise additional grant and philanthropic funding to support future discovery and development efforts.

While the inability to access venture capital in the early discovery phase is a potential drawback of the open science model, it is helpful to note that the annual global research spend for biomedicine and drug discovery is approaching ~$300B ^45^, of which venture capital is only ~$10B ^46^. In one view, while the open science structure of M4K might forsake the opportunity to compete for $10B of venture funding, in providing wider access to public and philanthropic funding, it positions M4K better to access a far larger pool of capital.

2. *In-kind contributions to M4K*

It is often challenging for a traditional company to collaborate with academia because of protracted negotiations over the allocation of patent rights and prospective revenue streams. The open science structure of M4K and its affordable pricing positions has provided a solution, and has enabled M4K to rapidly enter into collaborations and access in-kind contributions from multiple organizations, including the Universities of Oxford, Toronto, Pennsylvania, and Houston, Tufts University, and the Children’s National Medical Center in Washington, DC. The forms of in-kind contributions from these various partners have included running experiments, providing scientific input, and reviewing both data and documents.

We have also had contributions from the private sector. Senior scientists from three different large pharmaceutical companies (Bayer, AbbVie, Boehringer Ingelheim) have provided advice to M4K, including knowledge gained from terminated internal drug discovery efforts. Contract research organizations and technology providers are contributing resources in-kind on a *pro bono* or reduced costs basis. Charles River Labs, a leading provider of drug discovery services, has an internal program that allows each employee to donate time to a charity of their choice. Its UK chemistry team has decided to allocate a significant amount of staff time to advance M4K’s chemistry program, and their pharmacology and biology colleagues have provided drug discovery expertise free of charge. Reaction Biology Corp., a provider of screening services, has donated its services to help M4K test the potency and selectivity of newly synthesized compounds. 

3. *Knowledge generation for the public good*

M4K is also proving attractive to public funders, likely because the company shares its ongoing science, and thus generates freely available knowledge for the community – a core aim of public science funders. The main vehicle for knowledge dissemination is M4K’s monthly team meetings, which are live-streamed and then made permanently available on YouTube. These meetings discuss ongoing and prospective science, including chemistry plans. Ancillary consequences of these open drug discovery meetings are that they create prior art and thus freedom to operate for M4K. They also attract new collaborators to its program.

M4K also benefits from collaborations with the OpenLabNotebooks efforts of the Structural Genomics Consortium. In this project, three collaborating scientists at the SGC at the University of Oxford communicate their progress regularly by publishing their lab notes on the internet. These contributions provide the community up-to-date access to the science that drives M4K, including structural biology of ALK2 and related kinases, cell-based assays and screening methods. These scientists also place their structural datasets into the Protein Data Bank. And while M4K is working diligently to disseminate its pre-clinical drug discovery information, there is room to improve as the information is not standardized nor as findable or accessible as it could be. This is in large part due to the fact that there is no community-accepted data repository for pre-clinical drug discovery information. M4K is working to develop such a repository so that it can be used to openly share all of its drug discovery datasets, as well as datasets from other open science drug discovery companies.

4. *The future: open science and clinical development*

As M4K moves beyond its early-stage drug discovery efforts, it intends to continue to openly share its science, to crowdsource solutions, and to solicit public scrutiny of its work to improve scientific output in the later stages of drug development. For example, it intends to share clinical trial protocols and analysis plans for public comment, and also release analysable datasets and associated metadata as soon as practicable after clinical study unblinding (while respecting informed consent of trial participants and appropriately de-identifying any personally-identifiable information).

## Government can encourage open science companies committed to affordable pricing through policy changes

Although M4K is pursuing a rare disease indication, we believe its open science model does not have to be limited to this area and could be used to discover innovative new medicines for larger markets. And though M4K has identified a viable path forward in the current funding and regulatory environments, a few policy changes could encourage more companies to adopt this promising new business model.

First, government and philanthropic funders could strategically channel more of their translational funding programs into open science drug discovery consortia and companies. Although it is not customary for government to provide support for science carried out within a corporate structure, in open science companies, the objectives are aligned with the public interest: knowledge generation and affordable pricing. These companies should be eligible to compete for public funds.

Regulators could also create specific infrastructure and incentives for open science drug development, while leaving current proprietary pathways in place. For example, national regulators could collaborate to develop an open drug development data repository to catalyse open projects. This is not unlike the new SPARK program initiated by the Pew Charitable Trust for antibiotic drug discovery ^47^. A jointly-developed repository of this nature could ensure ready accessibility of preclinical and clinical data for governments to use in marketing authorization and reimbursement decision-making processes. To incentivize the repository’s use, an open developer that deposited a preclinical or clinical dataset could be entitled to a reasonable period of protection against competitive use of the dataset during the timeframe *prior to* approval of the open developer’s marketing application. 

In the interest of encouraging more transparency in the drug discovery and approval process, regulators could also offer an exclusivity period extension for openly developed drugs that gain marketing approval, similar to the paediatric study, new indication, and GAIN Act extensions discussed above. To obtain this ‘open science extension’, a sponsor could be required to (i) demonstrate that it has diligently made its preclinical and clinical data publicly available via the open drug development data repository, (ii) provide a certification that it has not filed for patents, rendering the sponsor ineligible to list patents for the drug product in the FDA Orange Book or equivalent registries; and (iii) enter into an agreement with the relevant health technology assessment or drug procurement agency to set an affordable price ceiling for the medicine as a *quid pro quo* for the extended exclusivity entitlement.

Finally, while most of the world’s major drug product markets provide regulatory data exclusivity regardless of the public availability of a sponsor’s data, other countries only adhere to the World Trade Organization Trade-Related Aspects of Intellectual Property (“TRIPS”) agreement, which merely requires member states to protect “undisclosed” test data. Formally, competitors in countries that limit protection to “undisclosed” data could seek to register identical drugs based on a sponsor’s open data. This creates a perverse incentive for companies to maintain secrecy for as long as possible in all jurisdictions, undermining efforts to encourage more sharing of trial data ^26^. And while it may be good public policy in low- and middle-income countries to limit data exclusivity protections broadly, perhaps there is an argument to consider implementing them exclusively for open science companies committed to affordable local pricing. Specifically, these countries could extend data protection to sponsors of innovative new drugs whose data have been made public, at least where the sponsor has pursued an open, patent-free path to market for a medicine that addresses local needs at a reasonable price.

## Perspective

In summary, with M4K, open drug discovery has transitioned from a theoretical notion to a real-world test of the concept. Promising progress on many fronts - scientific, financial, and community participation - augers well for the success of the model. All players in the system, including governments and regulators, should consider supporting open science drug discovery as a commercially viable business mechanism to invent new, and affordable, medicines.

## Disclaimer

The views expressed in this article are those of the authors. Publication in Wellcome Open Research does not imply endorsement by Wellcome.

## Data availability

No data are associated with this article.
