# Supplementary material for: Ideation and implementation of an open science drug discovery business model – M4K Pharma
Source: Wellcome Open Res. 2018 Dec 6;3:154. [Version 1] doi: 10.12688/wellcomeopenres.14947.1 (PMC6346698; doi:10.12688/wellcomeopenres.14947.1)
Supplement: Peer review history

# Peer review: Referee response for version 1

**DOI:** 10.21956/wellcomeopenres.16301.r34401

**Published:** 2019-01-23

**Authors:** Bernard Munos

**Affiliations:** 1 FasterCures, A Center of the Milken Institute, Washington, DC, USA

For the last two decades, the open-source model has steadily gained acceptance in biomedical research and drug R&D. There is now a large number of initiatives and organizations that have embraced its principles, and are offering it as an alternative to the proprietary research models that have dominated the field for much of its existence. Predictably, its growing popularity has met with challenges and pushbacks. They fall in three categories:

1. Open-source is anti-patents, and patents are essential to entice businesses to engage in drug R&D, and investors to support them. Therefore, open-source is a non-starter.
2. If open-source is so great, where are the drugs that it has produced?
3. Perhaps it fits somewhere in the ecosystem -- and it is unclear where that is -- but certainly not as an alternative to conventional drug R&D

 

 The manuscript addresses these challenges.

 On the first item, the authors point out that in areas that make up most of the pharmaceutical market ("US, EU, Singapore, Japan, Australia, Taiwan, and South Korea"), regulators offer alternative ways to protect IP and to provide incentives, such as various types of regulatory exclusivity (Fig 1), and priority review vouchers (PRVs).  They go on to show that such non-IP-driven protections and incentives compare favorably with patents with respect to duration. They also enjoy advantages with respect to cost, administrative management, and simplicity, since they preclude the burdensome patenting process with its legal, translation, filing, maintenance, and prosecution fees. If this is true, why the hoopla?

 The authors are correct. In fact, industry loves regulatory exclusivities and PRVs for the reasons stated. Yet it remains deeply attached to patents:

- It sees them as an effective bulwark against attempts to water down intellectual property
- It sees litigation costs as offering a degree of additional protection since companies can outspend challengers
- It also see them as the currency that enables the transaction of assets during drug development, by giving them value

Some of these objections, especially the first one, tend to be generational. They disregard the benefits from widespread knowledge-sharing because companies, which historically have had little exposure to it have tended to be dismissive of its value. Yet, as this value is documented by big science initiatives (e.g., HGP and derivatives); the output of open-source models (e.g., SGC, Critical Path Initiative); and the now routine requirement of many funders that the results of the research they support be made immediately available to the scientific community, opposition to open-source has eroded.

 The third objection, however, that IP enables an innovation market that matches assets with the companies most motivated to develop them, deserves more attention from the authors. Some scholars, such as CIP@Gothenburg and its many followers, have argued that IP is just a device to create value, and the holders of that IP can do whatever they want with it -- hold it, sell it, donate it, or put it in the public domain). So, it is not incompatible with open-source. Perhaps, the resolution of that debate hinges on regulators offering additional ways to recognize value earlier. The FDA's Breakthrough Therapy Designation has been a step in that direction, and companies and investors have been quick to embrace it. It is most often granted based on phase 2 data, but could conceivably be awarded sooner if drug developers can show evidence of superior treatment response along with safety.

 The authors then describe their experience with M4K, a startup designed to show that open-source can deliver novel therapies as well as or better than conventional R&D. M4K is owned by the Agora Open Science Trust, and seeks a treatment for DIPG, a rare and fatal pediatric brain cancer with no treatment. It claims no patent, puts immediately all its data in the public domain, but restricts the right to use it for regulatory purposes (to thwart misappropriation by potential competitors). It is funded by grants from government, foundations, and donors who adhere to its open-source commitment. It conducts its works through scientists and organizations that share the same ethos (e.g., Charles River Labs, Ontario Institute for Cancer Research). Some pharma companies have also shown interest in participating. Any drug that it produces will be licensed to a partner who will receive the right to use the data package for securing approval upon the condition that it will make the drug affordable. After one year of operation, M4K has ALK inhibitors in lead optimization.  Most of its support has come from non-profit sources, but several large pharma companies (Bayer, AbbVie, Boehringer Ingelheim) have made in-kind contributions.

 These results are interesting and encouraging, but fall short of a full validation of the model, which will only happen when a drug has been approved. Yet, one can make several comments about challenges that the model might experience:

- How scalable is it? Can we rely on non-profit sources and benevolent scientists to produce dozens of drugs annually? How far can we go before we dry up the pool of such talent. Perhaps surprisingly, there is ground for optimism that the authors should explore. The rare disease community has experimented with many low-cost models of drug development -- some of which have yielded drugs -- that are not radically different from M4K. There is also lots of talent trained by industry but now idled or retired, which would love to work on meaningful projects. And there is a venture philanthropic community which does not always find the outlets it seeks for its generosity. What's missing is a clearinghouse that can match needs with resources -- perhaps an endeavor whose time has come.
- M4K's drug candidates are small molecules. This is maybe inevitable since they must penetrate the brain. But small molecules is a tough domain to demonstrate superiority over pharma because of the vast store of tacit knowledge that the industry has accumulated when it comes to things like improving ADME or mitigating toxicity. It would have been easier to "compete" with different drug candidates (e.g., biologicals) where pharma does not have as much of a translational research advantage. But this may not have been an option for DIPG.

 

 This brings us to the last question, which is the role of open-source in the drug R&D ecosystem. Is it a replacement, an alternative, or a partner to pharma? It would be interesting to get the authors' views on this. Clearly some data about costs and timeline would help understand how M4K compares with traditional pharma, but the early stage of M4K's research may make this premature. Still, as uninformed opinions are being floated that we can, for instance, fix the drug pricing problem by getting the government into drug manufacture, or other dubious schemes, It would be interesting to hear from the authors how they see open-source fitting in the ecosystem, and contributing to making drug innovation affordable, in view of their experience with M4K.

I have read this submission. I believe that I have an appropriate level of expertise to confirm that it is of an acceptable scientific standard.

# Peer review: Referee response for version 1

**DOI:** 10.21956/wellcomeopenres.16301.r34396

**Published:** 2019-01-23

**Authors:** Matthew H. Todd

**Affiliations:** 1 University College London, London, UK

This paper by Morgan, Roberts and Edwards is a clear, important contribution on many levels that is suitable for publication with a few minor modifications. It deserves to be widely read and its implications understood.

 It is refreshing to see this specific subject matter so squarely addressed, and with an evolving example. Many of the technological barriers to working openly are quickly being solved. Alleviating the psychological worries of working openly is more of a challenge. This article correctly passes quickly over many of these issues, however, and instead focuses on the key economic objection that is often heard. As a practitioner of open source drug discovery, I have experience in receiving the question "how can you square openness with a sustainable economic model?" There is no one answer, just as there is no one medicine, or disease. Many interested and well-qualified people working to solve problems with the way we discover and develop new medicines (including my colleague Mariana Mazzucato, who I see has also written a review of this paper) warn against exclusivities as not being in the spirit of a necessary change of culture, and bundle them with patents as monopolies that interfere with equitable pricing (e.g. Section 4.2 of the People's Prescription report). Whether one agrees with this position or not, the value of this paper in the ongoing discussion is the thesis that exclusivities can conversely be seen as enabling of open research, in this case of providing an economic model for drug discovery that is independent of the patent system, meaning, crucially, that the R&D can be freely shared. This is a radical change in culture, if it can be made to work. As the authors note, it may in fact be worth enforcing exclusivity arrangements *more* aggressively for open science projects (with the realistic assumption that such projects are wedded to affordability) as a means to help them flourish. A missing citation here is Marden's relevant (but seldom-cited) paper ^1^ which includes the paragraph "For example one could introduce a new form of data exclusivity... This would allow the drug developer a fixed term to recoup some of its investment." (Minn. J. L. Sci. Tech. 2010, 11, 217)

 The paper makes an excellent general argument, and it is exciting that a currently-operating private-sector initiative, M4K, is used as an example. The authors make no claim that this is *the* example, which is appropriate. For example, the authors lay out the future strategy as one relying on a licensing strategy "to one or more partners capable of bringing the medicine to patients" and there are other options here (it might be appropriate to point the reader to the general discussion of this from my, and others', open source pharma roadmap paper ^2^). It is interesting that the authors are not optimistic about the value of venture capital investment as part of the investment portfolio (something I have argued for) and I would be interested in 1-2 sentences to account for this pessimism. Is there a structural problem, or is it distaste at the likely terms? There are (currently nebulous but nevertheless interesting) arguments, for example, that Blockchain technology could permit a series of locked-in contracts that would enable easier mixed investment, with individual terms for individual investors. If contracts can be bespoke, why limit the source of money?

 The comments made about the quality inputs received from the community in an open source research project ("A Partnering Strategy...") are interesting - others, including me, have witnessed the same effect and a reference to these examples would not be inappropriate to illustrate that these are not accidents, e.g. the substantial and counter-intuitive involvement of the private sector, the involvement of federated student groups and the value of live-online strategy meetings ^3^. Similarly the comment about open notebooks as the bedrock of the technical platform could usefully cite other experiences in this area (e.g. Labtrove or Chemotion), and the mention of databases could cite existing ways of sharing related data that precede the new SPARK initiative, such as ChEMBL (even though ChEMBL does not currently prioritize clinical data).

 The paper is arguing the case, via a specific example, for an "alternative" to the more traditional patent-based pharma model. The most valuable feature of the article is a reminder of the distinctness of the patent system from the various forms of data and market exclusivity. It is surprising how little-known this is in the general drug discovery community. What is particularly valuable in this article is the Table of examples of medicines either without patents or where patents expired before NCE exclusivity. The authors are to be congratulated on this valuable, citable resource that will help move the community discussion forward.

 The sentence "Finally, while most of the world’s major drug product markets provide regulatory data exclusivity regardless of the public availability of a sponsor’s data" for me is crucial. It would be helpful to know if the corollary could be stated explicitly - that the real-time public availability of clinical data will be no impediment to the granting of exclusivity in certain jurisdictions, or whether this statement is not yet completely clear. Have there ever been examples of exclusivities being granted where the relevant data were all public domain? Does e.g. the FDA have a stated position? The uncertainty over this issue (arising from the language used in TRIPS around "protecting" undisclosed data) may be sufficient to deter future investors and it would be helpful to be shot of it.

I have read this submission. I believe that I have an appropriate level of expertise to confirm that it is of an acceptable scientific standard.

## References

[1] MardenE: Open Source Drug Development: A Path to MoreAccessible Drugs and Diagnostics?. *Minnesota Journal of Law, Science*.

[2] BalasegaramMKolbPMcKewJMenonJOlliaroPSablinskiTThomasZToddMHTorreeleEWilbanksJ: An open source pharma roadmap. *PLoS Med*.14(4) : 10.1371/journal.pmed.1002276 e1002276 10.1371/journal.pmed.1002276 28419094PMC5395155

[3] WilliamsonAEYliojaPMRobertsonMNAntonova-KochYAveryVBaellJBBatchuHBatraSBurrowsJNBhattacharyyaSCalderonFCharmanSAClarkJCrespoBDeanMDebbertSLDelvesMDennisASDerooseFDuffySFletcherSGiaeverGHallyburtonIGamoFJGebbiaMGuyRKHungerfordZKirkKLafuente-MonasterioMJLeeAMeisterSNislowCOveringtonJPPapadatosGPatinyLPhamJRalphSARueckerARyanESouthanCSrivastavaKSwainCTarnowskiMJThomsonPTurnerPWallaceIMWellsTNWhiteKWhiteLWillisPWinzelerEAWittlinSToddMH: Open Source Drug Discovery: Highly Potent Antimalarial Compounds Derived from the Tres Cantos Arylpyrroles. *ACS Cent Sci*.2016;2(10) : 10.1021/acscentsci.6b00086 687-701 10.1021/acscentsci.6b00086 27800551PMC5084075

# Peer review: Referee response for version 1

**DOI:** 10.21956/wellcomeopenres.16301.r34402

**Published:** 2019-01-17

**Authors:** Mariana Mazzucato

**Affiliations:** 1 Institute for Innovation and Public Purpose (IIPP), University College London (UCL), London, UK

**Overview**

 In this open letter, the authors describe the experience of M4K pharma, which operates with an open science model for drug discovery.

 It opens with an explanation of the problem faced by the current drug discovery system, especially the lack of productivity. Highlighting the “proprietary generation and use of knowledge” as a key cause of the inefficiency in the collaborative innovation process, the authors argue that an open science model without patent restrictions is the necessary way forward. They further explain that regulatory exclusivity, which compares favourably to patents (in terms of length of protection, cost and the provision of regulatory certainty) and has already been widely applied in the real-world setting, can be used as the mechanism to protect intellectual property and incentivise innovation in lieu of patents. Then, the authors focus on M4K pharma as an example of a viable open science business model that uses regulatory exclusivity to protect intellectual property without hindering knowledge dissemination, and one that is structured to prioritise access over profit for its organisational goals. The authors report that M4K has demonstrated feasibility and success in attracting public and private resources to support open science, and propose that given stronger support from governments and regulators, the M4K model can be scaled up and replicated in other disease areas outside M4K’s expertise.

 Overall, the content of the article receives a favourable review from the reviewer. Appropriate to the Open Letter format, the article introduces the important and exciting work of M4K, a very laudable effort that shows the world 1) pharma innovation can be done differently and at the same time done well, and 2) open science is an achievable and practical idea, and it is high time to drop both nay-saying and paying lip-service to the idea and just put it in practice. The language of the article is accessible for the lay reader.

 However, the article has insufficiencies in parts in its current form that weaken its emphasis, flow and strength of arguments.

**Specific recommendations**

1. The article needs an introduction to explicitly set out its purpose and rationale. Crucially, it needs to explain what M4K Pharma does and the rationale for the choice of its disease speciality right at the beginning. It should also briefly explain why M4K can be a good example of the open science business model.
2. The article has too many sections, which interrupts the flow of the piece. For example, sections that explain the context of drug discovery and establish need for an open science business model, including “The drug development business model” and “Open science and the discovery of drug targets”, can be combined under one single heading of, for instance, ‘The need for an open science drug development model’. Similarly, sections that explain the use of regulatory exclusivity can be grouped together and made more succinct.
3. The relevance of applying regulatory exclusivity to the M4K model has to be explicitly established. The article convincingly argues that the protection of intellectual property is the key concern for any open science model. From a legal-economic perspective this is true, but there are other factors at work too, such as technological infrastructure, organisation, politics and culture. The encouraging progress made by M4K described the article in a way shows that having an alternative mechanism to protect intellectual property and at the same time permits open science is a necessary but insufficient condition for an open science business model to work. Is the chosen emphasis of this article on regulatory exclusivity simply a general assessment, or is it to do with the fact that this is fundamental to M4K’s business model? Either way, recognising that there are other factors that can make open science difficult (doesn’t have to be long – perhaps an extra sentence or two just to outline other factors) will give the reader a better sense of the field. Moreover, having dedicated a significant proportion of content to explaining the context and key concepts, the article needs to explicitly address why the foregoing is important to the experience of M4K (because the article is about M4K after all).
4. The description of how regulatory exclusivity works as a non-patent approach to protecting intellectual property is somewhat partial, and slightly biased towards its advantages. First, the relationship between regulatory exclusivity and patent is not clearly explained. Regulatory exclusivity is a statuary provision and is often used in parallel with patents; the two are not mutually exclusive. The article can be clearer on that, especially when in the abstract it describes regulatory exclusivity is used “in lieu of” patents, which may seem to hint at mutual exclusivity to the lay reader. This leads to the second point, that the comparison between regulatory exclusivity and patents misses the point why patents are preferred. The comparison here mainly concerns the time span of the term of protection granted by patents vis-à-vis regulatory exclusivity, which is useful, but it does not represent the full picture. On the one hand, there’s a question of generalisability of the analysis. Regulatory exclusivity tends to be longer in developed economies such as the US and the EU, and it tends to be stronger for special indications exemplified by orphan diseases (presumably part of the reason why M4K chose rare disease to test its business model--rare indications can play to the advantages of regulatory exclusivity more than others). On the other hand, while both mechanisms are used to delay entry of generics, patents – as rightly noted in the article – allow for a greater scope of secrecy and flexibility (though this comes with a higher cost and introduces a higher degree of uncertainty); but this is exactly the reason why they are a key tool for pharmaceutical company to maintain and prolong monopoly (this aspect is discussed in detail in, for example, " The people's prescription: Re-imagining health innovation to deliver public value"). Therefore, third, the article can benefit from better situating the non-patent-based approach described here in an innovation system that overridingly favours the use of patent as a tool for rent-seeking. The article has done a good job in highlighting the merits of only using regulatory exclusivity, but for the reader to appreciate the wholistic picture, it needs to acknowledge its drawback vis-à-vis patents in terms of profit generation – the life and blood of the pharmaceutical industry – and thus the potential resistance an alternative, non-patent business model is likely to encounter.
5. The limitations of the M4K model is not sufficiently discussed. While positive experiences of collaborating with the private sector highlighted in the article are highly positive and encouraging, the implicit limitations and challenging aspects for this model to disrupt the status quo are reflected in the type of inputs from the private industry partners at this stage, which are limited to in-kind contributions under the corporate social responsibility framing. But the ability to bring in private actors’ cash to the game is absolutely crucial if the open science model proposed here is to access the ~$300bn potential in the global research spend for biomedicine noted in the article, as much of the cash will concentrate in the private sector. In light of the analysis made in point 4 of this review, there will certainly be challenges going forward in order to broaden the application of M4K’s experience to areas outside rare disease indication and outside developed economies. Based on M4K’s experience and future plans, what are the possible measures to overcome its limitations?
6. Given the above, the article can draw a more nuanced conclusion than the existing one, namely “Although M4K is pursuing a rare disease indication, we believe its open science model does not have to be limited to this area and could be used to discover innovative new medicines for larger markets”. Before covering broader policy recommendations that can encourage open science overall, it can first draw direct lessons and implications that are more pertinent to M4K’s example.
7. The article needs a proper conclusion section to summarise a balanced perspective on the experience of M4K and open science models in general.

I have read this submission. I believe that I have an appropriate level of expertise to confirm that it is of an acceptable scientific standard, however I have significant reservations, as outlined above.
